# Association Between Environmental Health, Ecosystem Vitality, and Early Childhood Caries

**DOI:** 10.3389/fped.2020.00196

**Published:** 2020-05-19

**Authors:** Morenike O. Folayan, Maha El Tantawi, Robert J. Schroth, Arthur M. Kemoli, Balgis Gaffar, Rosa Amalia, Carlos A. Feldens, ECCAG

**Affiliations:** ^1^Department of Child Dental Health, Obafemi Awolowo University, Ife, Nigeria; ^2^Department of Pediatric Dentistry and Dental Public Health, Faculty of Dentistry, Alexandria University, Alexandria, Egypt; ^3^Department of Preventive Dental Science, Dr. Gerald Niznick College of Dentistry, Rady Faculty of Health Sciences, University of Manitoba, Winnipeg, MB, Canada; ^4^Department of Paediatric Dentistry and Orthodontics, University of Nairobi, Nairobi, Kenya; ^5^Preventive Dental Sciences Department, College of Dentistry, Imam Abdulrahman bin Faisal University, Dammam, Saudi Arabia; ^6^Department of Preventive and Community Dentistry, Faculty of Dentistry, Universitas Gadjah Mada Yogyakarta, Yogyakarta, Indonesia; ^7^Department of Pediatric Dentistry, Universidade Luterana Do Brasil, Canoas, Brazil

**Keywords:** environmental health, ecosystem vitality, environmental performance index, early childhood caries, air pollution

## Abstract

**Background:** Environmental issues lead to serious health problems in young growing children. This study aims to determine the association between a country's level of environmental health, ecosystem vitality, and prevalence of early childhood caries (ECC).

**Methods:** This was an ecological study. The data for the explanatory variables—country-level environmental performance index (EPI), environmental health, and ecosystem vitality—were obtained from the Yale Center for Environmental Law and Policy. The outcome variables were country-level prevalence of ECC in 0- to 2-year-old and 3- to 5-year-old children. The country EPI, environmental health, and ecosystem vitality were matched with country ECC prevalence for 0- to 2-year-olds and 3- to 5-year-olds for the period of 2007 to 2017. Differences in the variables by country income level were determined using ANOVA. Multivariate ANOVA was used to determine the association between ECC prevalence in 0- to 2-year-olds and 3- to 5-year-olds, and EPI, environmental health, and ecosystem vitality, adjusting for each country's per-capita gross national income.

**Results:** Thirty-seven countries had complete data on ECC in 0- to 2-year-old and 3- to 5-year-old children, EPI, environmental health, and ecosystem vitality scores. There were significant differences in ECC prevalence of 0- to 2-year-olds and 3- to 5-year-olds between countries with different income levels. Also, there were significant differences in EPI (*P* < 0.0001), environmental health score (*P* < 0.0001), and ecosystem vitality (*P* = 0.01) score by country income levels. High-income countries had significantly higher EPI scores than did low-income countries (*P* = 0.001), lower-middle-income countries (*P* < 0.0001), and upper-middle-income countries (*P* < 0.0001). There was an inverse non-significant relationship between ECC prevalence and EPI in 0- to 2-year-olds (*B* = −0.06; *P* = 0.84) and 3- to 5-year-olds (*B* = −0.30; *P* = 0.50), and ecosystem vitality in 0- to 2-year-olds (*B* = −0.55, *P* = 0.08) and 3- to 5-year-olds (*B* = −0.96; *P* = 0.02). Environmental health was directly and non-significantly associated with ECC in 0- to 2-year-olds (*B* = 0.20; *P* = 0.23) and 3- to 5-year-olds (*B* = 0.22; *P* = 0.32).

**Conclusions:** There was a complex relationship between various indicators of environmental performance and ECC prevalence. The association with EPI and ecosystem vitality was inverse whereas the association with environmental health was direct. Only the inverse association with ecosystem vitality in 3–5 year old children was significant. There may be higher risk of ECC with greater economic development, industrialization, and urbanization, while better ecosystem vitality may offer protection against ECC through the rational use of resources, healthy life choices, and preventive health practices.

## Background

There are few studies linking environmental factors and early childhood caries (ECC), which is caries in children <72 months of age with one or more decayed (cavitated and non-cavitated) or missing tooth due to decay or filled primary tooth surfaces (1). There are reports of possible associations between climate change and oral health (2, 3). Exposure to airborne pollution is the fourth leading cause of premature death globally, and ~5.5 million people die prematurely from air pollution each year (4). Air pollution also reduces the quality of one's overall health (5, 6).

Air pollution consists of a mix of pollutants of which particulate matter is the deadliest (7). Ambient particulate matter pollution is associated with pneumonia, stroke, ischemic heart disease, chronic obstructive pulmonary disease, and lung cancer (8–10). Particulate matter results from incomplete combustion of solid fuels, which are predominantly from biomass burning such as wood, crop wastes, charcoal, coal, and dung in households (11). Incomplete combustion in these households can produce fine particle concentrations up to 100 times higher than acceptable levels (7, 12). Reducing outdoor and indoor air pollution can bring substantial health and development benefits. There are, however, no studies on the association between ambient particulate matter pollution and caries, though evidence suggests plausibility for such an association. One study highlighted that exposure to carbon oxide ambient air contamination results in changes in the composition of saliva and teeth based on the severity of exposure (13). Exposure to lead can result in lead accumulation in teeth and may increase the risk for caries (14, 15), including ECC (2, 16).

Further, little is known about the direct association between unsafe water, unsafe sanitation, and ECC despite caries being referred to as a hygiene-related disease (17). One study indicated that perfluorodecanoic acid, an industrial surfactant found in drinking water systems, may disrupt the healthy development of enamel and increase its susceptibility to caries, including ECC (18). Another study showed a lack of association between ECC and water and sanitation as measures of poverty (19). Alternatively, Folayan et al. reported that good water collection and storage practices are associated with good oral hygiene in adolescents and young persons, 10–24 years old (20). Unsafe water also makes individuals resort to drinking more bottled beverages, most of which are high in sugar (21). More recently, there has been increasing interest in how the ecosystem vitality (biodiversity and habitat, tree cover loss, fisheries, climate and energy, wastewater treatment, and sustainable nitrogen management) affects health and well-being.

The evidence for a relationship between ecosystem vitality and oral health are few. The link between the environment and ECC can be explained through biological and/or behavioral pathways. Unhealthy environments increase the risk for malnutrition (2) and, thus, possibly ECC (22). These links are reinforced by economic influences that increase the risk for health problems, including ECC (known to be a disease of the socially disadvantaged) in persons who are socially disadvantaged. Economically vulnerable individuals are less able to mobilize private resources to cope with the impact of a poor environment, especially in politically fragile states (23). Thus, countries with poorer ecosystems and environmental health are less able to encourage the investments, financial growth, and working opportunities that can increase income per individual and economic development (23). Countries with better gross national income are better able to improve their ecosystems and environmental health (24).

Understanding the links between environmental performance and how community ecosystem vitality affects oral health may have implications for planning public health responses to ECC. This study aims to determine the association between country-level environmental health, ecosystem vitality, and ECC prevalence. We hypothesize that the prevalence of ECC will be higher as environmental health and ecosystem vitality get poorer.

## Methods

This was an ecological study. The explanatory variables were country-level data on Environmental Performance Index (EPI) and its two main components: environmental health and ecosystem vitality. The outcome variables were the prevalence of ECC in 0- to 2-year-old and 3- to 5-year-old children.

### Environmental Performance Index

This index, developed by the Yale Center for Environmental Law and Policy, ranks countries on their performance on environmental issues. It is a composite index formed by aggregating 24 standardized, weighted indicators under 10 domains representing two areas: environmental health, which measures threats to human health, and ecosystem vitality, which measures natural resources and ecosystem services. The weights used to calculate the EPI are shown in Appendix Table 1. Skewed indicators are log transformed, and all indicators are rescaled into 0 (worst) to 100 (best) scores to create the index. In the overall EPI, ecosystem vitality has greater weight (60%) than does environmental health (40%). The greatest weight in environmental health is air quality (65%), whereas in ecosystem vitality, the greatest weight is climate and energy (30%). Some indicators are not applicable for all countries, such as when countries are landlocked and have no fisheries (*n* = 44) and when countries have no forests (*n* = 30). In these cases, the weights are set to zero. Imputation is used to derive missing values for countries with no data (24). We used the 2018 data since it reflects on performance during the study period (2007 to 2017). Data are freely available through the website of the Yale Center for Environmental Law and Policy.

### Early Childhood Caries

The data on ECC prevalence were extracted from the World Health Organization's Country Oral Health Profile database and studies published and indexed in MEDLINE, Scopus, Web of Science, and Google Scholar covering the period 2007–2017. No language filter was applied. The retrieved data were used to calculate the ECC prevalence for each country by dividing the number of children affected by ECC by the number of children examined and multiplying by 100. We used the prevalence of ECC for 0- to 2-year-old and 3- to 5-year-old children. Further details were reported in our previous paper (25).

### Data Analysis

The datasets on EPI and ECC were matched by country. Countries were classified by income level according to the 2017 Gross National Income per Capita calculated by using the World Bank Atlas method (26) based on our previous finding of the association between global ECC prevalence and growth in per capita gross national income (25). The levels were as follows: low-income countries (LICs), $995 or less; lower-middle-income countries (LMICs), $996–3,895; upper-middle-income countries (UMICs), $3,896–12,055; and high-income countries (HICs), $12,056 or more.

Descriptive statistics were computed for the EPI, its two main components (ecosystem vitality and environmental health), and ECC in 0- to 2-year-old and 3- to 5-year-old children by income level, and compared using ANOVA. This computation was followed by post hoc pairwise comparison using Scheffe's test when a significant ANOVA was observed. IBM SPSS for Windows version 22.0 (IBM Corp., Armonk, N.Y., USA) was used to construct two models of multivariate ANOVA to assess the association between the outcome variables (ECC prevalence in 0- to 2-year-olds and 3- to 5-year-olds) and explanatory variables: EPI and environmental health score (Model 1) and ecosystem vitality score (Model 2) adjusted for income level. Regression coefficients (*B*), 95% confidence intervals (CI), *P*-values, and partial eta squared as measures of effect size were calculated. Significance level was set at 5%.

## Results

Thirty-seven countries had complete data on ECC in 0- to 2-year-old and 3- to 5-year-old children, EPI, environmental health score, and ecosystem vitality score (Appendix Table 2). Table 1 reveals that there were significant differences in ECC prevalence of 0- to 2-year-olds (*P* = 0.03) and 3- to 5-year-olds (*P* = 0.008) by country income levels. Children 0–2 years old (*P* = 0.04) and 3–5 years old (*P* = 0.02) in HICs had significantly lower ECC prevalence than did children in LMICs. There were no significant differences in ECC prevalence in 0- to 2-year-olds and 3- to 5-year-olds in countries with other income levels.

**Table 1:** Association between ECC prevalence, EPI, EPI components, and different income levels of countries included in the study.

	**LICs**	**LMICs**	**UMICs**	**HICs**	**All**	***P*-value**
*N*	2	11	10	14	37	
ECC prevalence in 0- to 2-year-old children, % (SD)	11.50 (8.86)^ab^	32.15 (18.55)^a^	26.83 (12.02)^ab^	16.33 (10.94)^b^	23.61 (15.20)	0.03
ECC prevalence in 3- to 5-year-old children, %(SD)	39.13 (2.65)^ab^	62.62 (20.33)^a^	58.36 (17.88)^ab^	37.02 (19.34)^b^	50.51 (21.8)	0.008
EPI—Score (SD)	47.56 (4.63)^a^	52.20 (10.18)^a^	58.20 (4.44)^a^	74.27 (7.36)^b^	61.92 (12.55)	<0.0001
Environmental health—Score (SD)	39.32 (8.03)^a^	50.37 (20.96)^a^	61.66 (13.42)^a^	90.22 (8.52)^b^	67.90 (23.21)	<0.0001
Ecosystem vitality—Score (SD)	53.05 (2.35)^ab^	53.42 (6.96)^a^	55.89 (6.55)^ab^	63.64 (9.17)^b^	57.93 (8.72)	0.01

a,b *different letters denote statistically significant differences among countries with different income levels*.

Table 1 shows that there were also significant differences in the EPI (*P* < 0.0001), environmental health score (*P* < 0.0001), and ecosystem vitality (*P* = 0.01) score by country income levels. HICs had significantly higher EPI scores than did LICs (*P* = 0.001), LMICs (*P* < 0.0001), and UMICs (*P* < 0.0001). HICs also had significantly higher environmental health scores than did LICs (*P* = 0.001), LMICs (*P* < 0.0001), and UMICs (*P* = 0.001). In addition, HICs had significantly higher ecosystem vitality scores than those of LMICs (*P* = 0.02). There were no significant differences in the ecosystem vitality scores among countries with other income levels, though the ecosystem vitality scores increased as country income levels increased.

The prevalence of ECC in 0- to 2-year-olds and 3- to 5-year-olds by ecosystem vitality scores were not significantly different between LICs and UMICs or between each of them and LMICs or HICs. However, the prevalence of ECC in LMICs was significantly higher than in HICs. The same differences were observed for ecosystem vitality. EPI and environmental health scores were significantly higher in HICs than in LICs, LMICs, and UMICs. There were no significant differences in the scores between LICs, LMICs and UMICs.

Table 2 Model 1 shows the association between the prevalence of ECC in 0- to 2-year-old and 3- to 5-year-old children and the EPI. The prevalence of ECC was inversely and non-significantly associated with EPI for 0- to 2-year-olds (*P* = 0.84) and 3- to 5-year-olds (*P* = 0.50). The results indicate that countries with one-point higher EPI had lower prevalence of ECC among 0- to 2-year-olds (B = −0.06) and 3- to 5-year-olds (B = −0.30), with a stronger association in older (partial eta squared = 0.01) than younger children (partial eta squared = 0.001). There was greater power to detect associations in 3- to 5-year-old children (power = 0.81) than in 0- to 2-year-old children (power = 0.63).

**Table 2:** Association between ECC and environmental performance indicators based on multivariate analysis of variance.

**Models**	**Explanatory variables**	**Percentage of 0- to 2-year-old children with ECC**			**Percentage of 3- to 5-year-old children with ECC**		
		**B (95% CI)**	***P*-value**	**Partial eta squared**	**B (95% CI)**	***P*-value**	**Partial eta squared**
Model 1	Environmental performance index	−0.06 (−0.72, 0.59)	0.84	0.001	−0.30 (−1.18, 0.59)	0.50	0.01
Model 2	Environmental health	0.20 (−0.13, 0.52)	0.23	0.05	0.22 (−0.22, 0.65)	0.32	0.03
	Ecosystem vitality	−0.55 (−1.17, 0.07)	0.08	0.10	−0.96 (−1.78, −0.14)	0.02	0.15

*The models are adjusted for income level*.

In Model 1, partial eta squared of income level was 0.16 for ECC in 0- to 2-year-old children and 0.15 for ECC in 3- to 5-year-old children. The power for ECC in 0- to 2-year-old children was 0.63 and 0.81 for 3- to 5-year-old children.

In Model 2, partial eta squared of income level = 0.19 for ECC in 0- to 2-year-old children and 0.20 for ECC in 3- to 5-year-old children. The power for ECC was 0.80 in 0- to 2-year-old children and 0.94 in 3- to 5-year-old children.

Model 2 shows the association between the prevalence of ECC in 0- to 2-year-old and 3- to 5-year-old children, environmental health, and ecosystem vitality. The association between ECC and the ecosystem vitality was stronger than the association between ECC and environmental health for 0- to 2-year-olds (partial eta squared = 0.10 vs. 0.05) and for 3- to 5-year-olds (partial eta squared = 0.15 vs. 0.03).

Environmental health was directly associated with ECC prevalence: countries with higher environmental health scores had higher ECC prevalence for 0- to 2-year-olds (*B* = 0.20) and 3- to 5-year-olds (*B* = 0.22). The association, however, was not statistically significant.

Ecosystem vitality, though, was inversely associated with ECC prevalence: countries with lower ecosystem vitality scores had higher ECC prevalence for 0- to 2-year-olds (*B* = −0.55) and 3- to 5-year-olds (*B* = −0.96). Only the association in 3- to 5-year-olds was statistically significant (*P* = 0.02). There was adequate power to detect associations among 0- to 2-year-old children (power = 0.80) and 3- to 5-year-old children (power = 0.94).

## Discussion

It is important to determine if there are relationships between the prevalence of ECC, environmental health, and ecosystem vitality, since these indicators differ by country. In the present study, we found inverse associations between the prevalence of ECC and the overall EPI and between the prevalence of ECC and ecosystem vitality. We also found a direct association between the prevalence of ECC and environmental health. The only statistically significant relationship was that between the prevalence of ECC in 3- to 5-year-olds and ecosystem vitality. The association with ecosystem vitality was stronger than that for environmental health. The effect sizes of the associations were small, as were the sample sizes. Our observations in this study may be early evidence of relationships between environmental variables and ECC.

In our study, EPI differed by income region. We found a reverse U-shaped curve for the relationship between ECC prevalence and country income level—for both the 0- to 2-year-old and 3- to 5-year-old age groups—with the highest ECC rates for LMICs/UMICs, and lower rates for LICs and HICs. The other environmental measures—EPI, environmental health, and ecovitality—appear to have a positive linear relationship with income. This relationship indicates that there may be a complex, non-linear relationship between ECC and environmental measures, which might be elucidated when more data are available for more countries, especially at more detailed administrative unit and/or individual levels. What our findings suggest is that in the LICs to LMICs/HMICs transition, there may be a positive association between increasing environmental measures and increasing ECC prevalence (mostly associated with globalization, urbanization and increased income leading to increased sugar consumption). In the LMICs/UMICs to HICs transition, there may be a negative association between improved environmental health and ECC prevalence (mostly associated with increased consumption of fluoridated water). Our finding that HICs had significantly higher EPI scores than did countries with lower income levels is consistent with the findings of a prior study that showed a positive correlation between EPI and country wealth (27). Though this cross-sectional ecologic design cannot provide evidence for causality, the literature indicates that countries with higher incomes can afford the resources to invest in the infrastructure required to support environmental performance in air quality, water, and sanitation for environmental health, biodiversity and habitat, climate and energy, and sustainable nitrogen management for ecovitality (27).

We found a small but direct association between ECC prevalence and environmental health and an inverse association between ECC and ecosystem vitality. Though the two components of the EPI (environmental health and ecosystem vitality) are positively correlated, there are distinct differences between them (27). As income increases, more resources are available to support environmental health. However, increased urbanization, consumption of natural resources, and resulting pollution challenge the vitality of the ecosystem (28). The two components of EPI are likely not a simple reflection of a country's wealth. The positive regression coefficients of environmental health in the present study may indicate a higher risk of ECC with greater economic development, industrialization, and urbanization. The possible protective effect of better ecosystem vitality on the risk of ECC may reflect a more rational use of resources accompanied by healthy life choices and preventive health practices.

The inverse association observed between the prevalence of ECC and ecosystem vitality requires further investigation. Ecosystem vitality is a composite of seven indicators: biodiversity and habitat, forests (tree cover loss), fisheries, climate and energy, water resources (wastewater treatment), agriculture (sustainable nitrogen management), and air pollution. The major contributor to the index is the climate and energy indicator (24). Though there is increasing interest in understanding the effect of greenhouse gases on the climate and how climate and energy affect health, there is a paucity of studies on the subject. We found an inverse relationship between ECC and ecosystem vitality with weak effect size. However, the pathway for this relationship is less understood. We posit that the same factors that contribute to improved ecosystem vitality—more rational use of resources with improved preventive health practices—lead to improved oral health care for children. Islam and Winkel (23) posit that improved ecosystems are associated with growth of assets and income, improved economic development, and improved general health of individuals (29). Climate change may also result in an altered effective dose of fluoride in water (30), with implications for cariogenic effects (31). Economies that are able to make more rational use of their resources would likely improve access to preventive oral care for children (25), including dental office-based procedures and health education to inform better choices, such as avoiding consumption of sugar-sweetened beverages. Similarly, countries with good wastewater treatment would be more likely to have public water supply fluoridation and potentially reduce the risk for ECC (32). Though these pathways suggest a link, not a cause–effect relationship, it is clear that the relationship between ECC and ecosystem vitality warrants further study.

Our study analysis does not indicate which of the seven indicators of ecosystem vitality play significant roles in the association between it and the prevalence of ECC in 3- to 5-year-olds. Though the major contributor to the index is the climate and energy indicator, and we can assume this may be a significant contributor, this needs to be explored further. Studies also need to explore for possible reasons why there was a significant association in 3- to 5-year-olds and not in 0- to 2-year-olds: may this reflect a dose-related relationship between ECC and eco-vitality? We shall explore this in further studies.

We did not observe a significant association between the environmental health score and ECC. The environmental health score for every country is a composite score of three indicators: air quality, water and sanitation, and exposure to heavy metals (specifically lead). Air quality constitutes 65% of the composite score (24). The lack of association between environmental health score and ECC was surprising since poor air quality has been associated with low birth weight, short gestation, wasting, underweight, and stunting (33–36). These outcomes increase the risk for enamel hypoplasia (37) and plaque retention, which are risk factors for ECC (38).

Our study has limitations. The environmental health scores for countries included in the study were higher than those for ecosystem vitality. This contrasts with a study by Wendling et al. (39) that showed higher scores for ecosystem vitality and lower scores for environmental health. The difference in our study may be attributed to the higher representation of HICs and UMICs. Our data, therefore, are skewed toward HICs and UMICs and may have limited generalizability with respect to global environmental performance. In addition, we acknowledge that, even within the same country, there is considerable variability among various social and economic gradients. Different relationships may be obtained if a greater number of countries with low/lower middle income are included. For example, if countries with poorer performance (resulting from less-regulated trade conditions and greater exploitation of resources) are included, the relationship between ECC and ecosystem vitality may become reversed. Also, the small sample size of countries with available data for ECC in 0- to 2-year-olds reduces the study power and makes it difficult for associations to be detected, especially if the exposure effect is small. In addition, not all the country-level data for ECC were generated from national surveys; a number were studies specific to administrative units, which was then generalized to the country. Thus, not all the country data used for this study may have truly represented the country. The cross-sectional nature of this study limits our ability to draw inferences about causal relationships independent of confounders of exposure-outcome association.

Like all ecological studies, there is the possibility of ecological fallacies resulting from the use of multiple aggregated data sets. We acknowledge that with ecological studies, correlations tend to be larger when associations are determined at the group, rather than the individual, level (40). Despite these limitations, this study enhances our understanding of the associations between environmental health and ECC and provides the first data on the association between ecosystem vitality and ECC. The study suggests that protection of the environment may affect the oral health of toddlers and preschool children, thus the need for environmental protection. This environmental protection is even more imperative in view of global climate change. More studies are required to understand the complex interactions between environmental health, ecosystem vitality, and ECC.

In conclusion, the study showed that there were inverse, non-significant associations between ECC prevalence and EPI and ecosystem vitality and direct association between ECC and environmental health. Only the inverse association with ecosystem vitality was significant in 3–5-year-olds. Our study hypothesis was, therefore, partly sustained. The study results suggest that there may be a higher risk of ECC with greater economic development, industrialization, and urbanization, while better ecosystem vitality may offer protection against ECC through the rational use of resources, healthy life choices, and preventive health practices.

## Data Availability Statement

Publicly available datasets were analyzed in this study. This data can be found here: https://epi.envirocenter.yale.edu/epi-topline.

## Author Contributions

MF and ME both conceptualized the manuscripts, developed the data analysis plan, developed the framework for the study report, and edited and finalized the manuscript. All other authors were involved with the review of the manuscript for intellectual inputs, finalization of the manuscript, and approved the manuscript for submission.

## Conflict of Interest

The authors declare that the research was conducted in the absence of any commercial or financial relationships that could be construed as a potential conflict of interest.
