# Supplementary material for: Association Between Environmental Health, Ecosystem Vitality, and Early Childhood Caries
Source: Front Pediatr. 2020 May 19;8:196. doi: 10.3389/fped.2020.00196 (PMC7248316; doi:10.3389/fped.2020.00196)
Supplement: Supplementary file 1 [file Table_1.docx]

**Appendix A**

**Table 1: Components and weights of the Environmental Performance Index (EPI)**

| Indicator | Weight | Components | Definition |
| --- | --- | --- | --- |
| Environmental health | | | |
| Air Quality | 65% | Household Solid Fuels | DALY rate from exposure to indoor air pollution from household use of solid fuels |
|  |  | PM_2.5_ Exposure | The average annual concentration of PM_2.5_ to which a citizen is exposed in μg/m3 |
|  |  | PM_2.5_ Exceedance | Weighted percentage of population exposed to annual concentrations of PM_2.5_ exceeding WHO guidelines at four levels: 10, 15, 25, and 35 μg/m3 expressed as % of population |
| Water & Sanitation | 30% | Drinking Water | DALY rate from lack of access or use of improved sources of drinking water. |
|  |  | Sanitation | DALY rate from lack of access or use of improved sanitation facilities |
| Heavy Metals | 5% | Lead Exposure | DALY rate from lead exposure |
| Ecosystem vitality | | | |
| Biodiversity & Habitat | 25% | Marine Protected Areas | Percent of a country’s Economic Exclusion Zone set aside as a marine protected area. |
|  |  | Biome Protection (National) | Percent of a country’s biomes in terrestrial protected areas, weighted by the prevalence of different biome types within that country |
|  |  | Biome Protection (Global) | percent of a country’s biomes in terrestrial protected areas weighted by the prevalence of different biome types around the world |
|  |  | Species Protection Index | Protected areas in relation to species distributions expressed as % of habitat |
|  |  | Representativeness Index | Extent to which a country’s protected areas are ecologically representative. |
|  |  | Species Habitat Index | Changes in the suitable habitats of species to provide aggregate estimates of potential population losses and extinction risk increases expressed as % of habitat |
| Forests | 10% | Tree Cover Loss | The five-year moving average of percent of forests lost. Forests are defined as land areas having ≥30% canopy cover measured as % of forested land |
| Fisheries | 10% | Fish Stock Status | Percentage of a country’s total catch that come from taxa that are classified as either over-exploited or collapsed |
|  |  | Regional Marine Trophic Index | The trends in the Regional Marine Trophic Indices of a country, or mean trophic level of the fish catch in each region of the country’s Economic Exclusion Zones (EEZs) |
| Climate & Energy | 30% | CO_2_ Emissions – Total | Intensity of CO_2_ emissions from the entire economy, as a blend of current-year intensity and a 10-year trend |
|  |  | CO_2_ Emissions – Power | Intensity of CO_2_ emissions per kilowatt-hour of electricity and heat, as a blend of current-year intensity and a 10-year trend |
|  |  | Methane Emissions | Intensity of methane emissions from the entire economy, as a blend of current-year intensity and a 10-year trend |
|  |  | N_2_O Emissions | Intensity of N_2_O emissions from the entire economy, as a blend of current-year intensity and a 10-year trend |
|  |  | Black Carbon Emissions | Intensity of Black Carbon emissions from the entire economy, as a blend of current-year intensity and a 10-year trend |
| Air Pollution | 10% | SO_2_ Emissions | Intensity of SO_2_ emissions from the entire economy, as a blend of current-year intensity and a 10-year trend |
|  |  | NO_X_ Emissions | Intensity of NO_X_ emissions from the entire economy, as a blend of current-year intensity and a 10-year trend |
| Water Resources | 10% | Wastewater Treatment | Percentage of wastewater treated, weighted by the connection rate of the population to the wastewater treatment system |
| Agriculture | 5% | Sustainable Nitrogen Management | Euclidean distance from an ideal point with optimal nitrogen use efficiency and crop yield. |
