# Supplementary material for: Association Between Environmental Health, Ecosystem Vitality, and Early Childhood Caries
Source: Front Pediatr. 2020 May 19;8:196. doi: 10.3389/fped.2020.00196 (PMC7248316; doi:10.3389/fped.2020.00196)
Supplement: Supplementary file 2 [file Table_2.docx]

**Appendix A**

**Table 2: Countries included in the study**

1. Australia
2. Belgium
3. Brazil
4. Canada
5. Chile
6. China
7. Colombia
8. Ecuador
9. Egypt
10. El Salvador
11. Finland
12. Germany
13. Greece
14. India
15. Indonesia
16. Israel
17. Italy
18. Japan
19. Kazakhstan
20. Kuwait
21. Kyrgyzstan
22. Mexico
23. Mongolia
24. Morocco
25. Namibia
26. Nigeria
27. Pakistan
28. Paraguay
29. Russia
30. Serbia
31. Sri Lanka
32. Sweden
33. Switzerland
34. Tanzania
35. Uganda
36. Ukraine
37. United States
